# Satisfied or not satisfied? Electronic health records system implementation in Ghana: Health leaders’ perspective

**DOI:** 10.1186/s12911-022-01998-0

**Published:** 2022-09-22

**Authors:** Priscilla Y. A. Attafuah, Patience Aseweh Abor, Aaron Asibi Abuosi, Edward Nketiah-Amponsah, Immaculate Sabelile Tenza

**Affiliations:** 1 Public Health Nursing Department, School of Nursing and Midwifery, University of Ghana, Legon, Ghana; 2 Department of Public Admin and Health Services Management, University of Ghana Business School, Legon, Ghana; 3 Department of Economics, School of Social Sciences, University of Ghana, Legon, Ghana; 4 School of Nursing Science, Faculty of Health Sciences, North-West University, Potchefstroom, South Africa; 5 School of Public Health, Faculty of Health Sciences, University of the Witwatersrand, Johannesburg, South Africa

**Keywords:** EHR, Electronic health records, Satisfaction, Quality of care, Health leaders

## Abstract

**Background:**

Electronic Health Records (EHR) has been espoused to be an innovation from the paper-based system, with benefits such as fast access to patient information thereby facilitating healthcare provider communication, healthcare continuity and improved quality of care. However, it is the extent of the quality of the electronic health records that determines the access to these stated benefits. The quality of health care records indirectly contributes to patient safety because inaccurate patient data can lead to improper diagnosis and consequently wrong treatment of patients. Most hospitals in Ghana, have recently transitioned into the EHR system, hence, there is a need to assess its accuracy, impact on workflow, staff training on usage, support from the EHR team, and the overall satisfaction of the EHR system. As health leaders are at the frontline of its implementation, their views on the challenges and successes of the EHR system are imperative.

**Method:**

This qualitative study sought to explore the views of the health leaders on the implemented electronic health records system in nine (9) hospitals within three (3) regions in Ghana. Following ethical approval, GHS-ERC:007/04/21, focus group discussions were conducted with a minimum of 10 hospital leaders in each facility. These included quasi, government and private hospitals. Data was collected between September and November 2021.

**Results:**

The study found poor quality of records, lack of involvement of frontline clinicians in the development of the EHR system, inadequate training of staff and limited workstations as some of the challenges associated with the use of EHR in hospitals. Health leaders were generally not satisfied with the EHR system.

**Conclusion:**

It is recommended that addressing inputs from end-users as well as circulating more computers will motivate EHR usage and acceptance. Provision of additional workstations for the various units and involvement of staff in the system development would be most prudent to enable health workers to accept the EHR system in improving the quality of care.

## Background

For decades, many hospitals and clinics in developing countries, have kept records of patients in hardcopy formats. In the current digital age, the electronic health records system has been introduced to ease data management such as the transfer of patient information from one unit to the other and in some cases, across hospitals. Electronic Health Records (EHR) as a storehouse of patient data kept in a digital format [6, 20] has been espoused to be an innovation from paper-based systems.

Various studies from developed countries have revealed that EHR, has many benefits, such as having the ability to reduce waiting times, keeping safe records of past medical events, as well as easy accessibility of patient data during referrals [5, 14, 15, 24]. However, it is the extent of the quality of the electronic health records that determines the fast access to patient information thereby facilitating health care provider communication, health care continuity and improved quality of care [4, 11, 20].

The quality of health care records indirectly contributes to patient safety because inaccurate patient data can lead to improper diagnosis and consequently wrong treatment of patients [18, 26]. The quality and accuracy of data stored in the EHR system make it easier for decision-making among health professionals in different departments [10]. For these reasons, most hospitals in Ghana, have recently transitioned into the EHR system, although not completely, certain categories of health facilities have adopted the system.

Currently, most teaching hospitals, regional hospitals, some district hospitals, and most private hospitals are using the EHR system. Depending on the finances of the health facilities, some computers/laptops are available in the units with EHR applications installed. Brands of EHR applications being used in health facilities across Ghana include; “CAREWEX EMR”, “HEALTH PRO ©”, “HIS”, “LHIMS” to mention a few. Some applications have been praised above others for varying reasons.

Despite the benefits stated by many authors, some facilities are not enjoying these benefits because of some barriers or challenges as enumerated by some studies regarding the use of EHR [5, 9, 13, 23]. These include inadequate computer skills, limited or non-available tools, limited funding, lack of technical basis, security and privacy issues, lack of technical expertise and, changes in workflow. In a critical review by Cho et al. [7], it was postulated that the acceptance or non-acceptance of the EHR system by health professionals is critical for attaining its use for improving the quality of care. Health leaders are the implementors of health policies and their reception of EHR will lead to its success or failure. Health leaders in this study referred to all categories of leaders in the facility, including fronline unit managers, departmental managers and executive top management. Hence, leaders in this study were those who were part of either the core management of health facilities or headed the various units or departments in the health facility as the case may be. Policy implementation and change management approaches encourage frontline managers including nurses to be involved in decision-making (Muthati et al. 2020). In Ghana, clinical and non-clinical heads are the ones who use the EHR often or directly oversee other staff who use it. As, these health leaders are at the frontline of EHR implementation, their views on the challenges and successes of the system are imperative [1]. Since the EHR system has been recently implemented, studies examining the satisfaction with its implementation in Ghanaianhospitals are scanty.

Hence, there is a need to assess the satisfaction of health leaders with the EHR from the end-user point of view. This study is also the first of its kind carried out on a large scale to the knowledge of the authors.

## Methods

### Study setting

This study was conducted in three regions in Ghana which were the Bono, Greater Accra and Upper East regions. Within each selected region, four hospitals were selected, to represent various categories of hospitals, including public, faith-based, private, and regional hospitals. Four hospitals (two, each from the Bono and Upper East regions) were finally excluded because they had not rolled out the EHR system in their facilities. In total there were nine (9) health facilities including public, faith-based, private, regional hospitals and one tertiary hospital. A tertiary hospital in the Greater Accra region was selected to represent all tertiary hospitals by convenience as well as it being the first tertiary hospital in Ghana. In all, nine health facilities were used.

### Study design

A qualitative study design was chosen to explore the views of hospital leaders on the current EHR system and their satisfaction with its implementation.

### Participant selection

A purposive sampling of hospital leaders was done to ensure that the selected participants have used the EHR system for at least one month. All leaders whose units had EHR initiated in their units were invited to partake. Being the head of a clinical or non-clinical unit was the inclusion criterion. Hence, all other healthcare workers who were not in leadership positions were excluded. Since leaders play an active role in facilitating change in an organisation, their views were most important for the study.

### Data collection instrument

A semi-structured Focus Group Discussion (FGD) guide, informed by literature and the study objectives and developed for data collection. The overall guiding question was “What is your assessment of the electronic health records system in this hospital?” The focus group discussion guide had sub-sections of probes guiding the discussion to cover identified challenges with the EHR system, transition from paper-based records to EHR, training on the EHR system, impact of EHR on workflow, support received from the EHR team and overall satisfaction with the EHR system.

A pilot study with one focus group was conducted in the Eastern region of Ghana, which is not among the study regions. This was necessary to ensure the appropriacy of content. There were no substantive changes necessary after piloting.

### Data collection

Data collection commenced from September till November 2021. Following ethics approval, by the Ghana Health Service Review Committee (GHS-ERC: 007/04/21), a team of six researchers with experience in qualitative data collection, contacted hospitals to seek permission to conduct the study. Upon receipt of permission, a list of unit heads with their contact numbers was provided by the in-service coordinators of the institutions. Participants were contacted via phone to request voluntary participation in the study. Some requested to have one-on-one interviews for fear of victimisation by colleagues should they be involved in the FGD. Hence one-on-one, as well as FGD, were organised in this study. Appointments were scheduled to conduct focus group interviews or individual interviews depending on the situation in each hospital, in a place and time convenient for the participants. On the day of data collection, researchers visited the facilities in pairs, one with the role of interviewing, the other with the responsibility of notes taking and managing the recording.

All interviews were conducted in English, in an enclosed space provided by the hospital to ensure privacy and minimize distractions. The interview began with an introduction to the study, and an explanation of the voluntary nature of participation. Participants were asked to sign a confidentiality agreement to protect all the information shared in the discussion and to put them at ease as they discuss their views. Following informed consent, the interviewer used the semi-structured interview guide to explore the views of hospital leaders on the implemented electronic health records system of the facility. Each interview lasted for about an hour, although the duration varied depending on the responses provided by the participants.

Interviews were recorded digitally and labelled with a FGD code. Immediately after the interviews, the interviewer wrote a synopsis of each interview to support data analysis. All audio recordings are kept on a password-protected computer to ensure confidentiality, and only the research team has access to the data.

### Data analysis

The interviews were transcribed verbatim. One researcher who was also part of the data collection for each region cleaned the data by reading each transcript while listening to the original recording. Following data cleaning, two researchers (PYAA and ST) analysed the data independently to achieve intercoder agreement. Inductive thematic analysis was used to analyse data. After independent coding, the first author who was involved in data collection, read through the codes to confirm that they depict what transpired during the interview discussions. Both researchers held coding meetings following the completion of data coding per region, these further contributed to an inter-coder agreement. The themes emerging from the codes were developed in these meetings and confirmed by both researchers and were further examined for similarities and differences across regions. The entire team approved the finalised themes.

### Trustworthiness and rigour

We applied Creswell's [8] criteria of trustworthiness in the study. The participation of two researchers in data analysis ensures the reliability of the findings. Attaching excerpts of narratives in the report writing to illustrate themes ensures confirmability. The iterative process of repetitive listening to the audio records during data cleaning allowed for prolonged engagement with the data, ensuring credibility. We read the field notes and used these as a reference to substantiate codes during the analysis of the rest of the data and to confirm the final generated themes.

## Results

A total of 100 health leaders who work in selected hospitals within the three (3) regions of the country participated in the FGD interviews. The hospital leaders who consented to participate were of different categories. Table 1 shows the detailed categories of participants.

**Table 1:** Categories of participants

Category of participant	Numbers
Nurse Manager (NM)	65
Pharmacy Manager (PM)	9
Accountants (AC)	18
Medical Assistant (MA)	2
Quality Assurance (QA)	4
Laboratory heads (LH)	6
Administrator (AD)	10
Health information officer (HI)	1
Physician Assistant (PA)	1

**Fig. 1:**
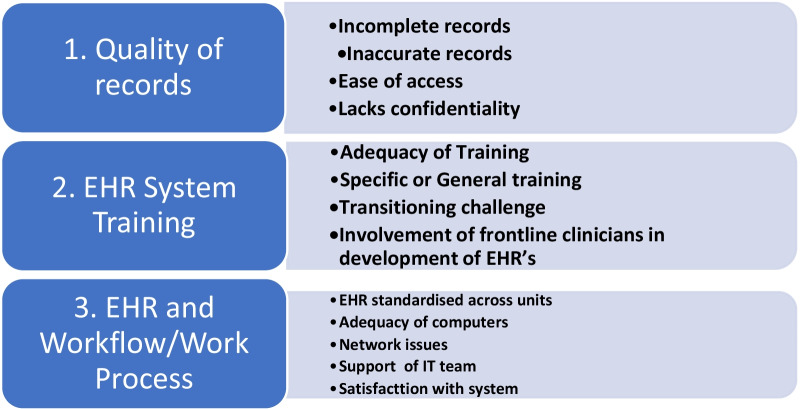
Themes and subthemes: Hospital’s Electronic Health Record (EHR) System. *Source*: Authors’ conceptualization

During analysis, we found three main areas through which health leaders measure their satisfaction or dissatisfaction with the electronic health records system in their various health facilities. We classified these areas as three major themes which had thirteen subthemes. The three main areas were: (1) Quality of records; (2) EHR System Training; (3) EHR and Workflow/Work Process. Details are shown in Fig. 1.

### Quality of records

Four subthemes emerged concerning the quality of electronic health records at various levels of health institutions in Ghana, namely: (1) Incomplete records, (2) Inaccurate records, (3) Confidentiality and (4) Ease of access. Each sub-theme is described below in detail.

#### Incomplete records

Participants expressed frustrations with incomplete records. These incomplete records from the professionals as narrated by NM11 were attributed to laziness as well as not having adequate knowledge in computing.

“The other aspect is that some are not so conversant with the computer and hence, patients delay in the consultation room for this reason they don't input everything there comprehensively. So it's just a little abbreviation and they are gone. I think it's sometimes laziness. You want to review what the doctor has done and its scanty information” (Bawku, NM11).

#### Inaccurate records

There were also complaints of inaccurate information being entered into the records. Some hospital personnel were either provided with wrong information by the clients or inserting the wrong details of clients in the computer, these were exacerbated by copying and pasting information from one record to another leading to double entries or incorrect entries on the patient’s file.

“There are times when incorrect information is copied and pasted from one patient record to another's records…” (HI); “… incorrect entries, we experience them a lot and sometimes also a replication of drugs. A drug that is supposed to be taken one week or for one month, you find the same drug entered twice by different people” (GAR-PM9); “… there was a patient we discharged … and then he was coming for review but when he came,… the system indicated “deceased” …” (KBT-NM29).

#### Ease of access

Participants often complained that the patient’s records were not easily accessible as they had to go through a series of navigations to get to them, they added that difficult access led to delays during consultation.

“… for instance, if I’m using the folder I can see let’s say three patients within let’s say 30 min but within that 30 min, I can only use to see one patient because of the system that we are using because typing is a bit slow to me it’s a bit slower than writing manually” (ASA-PA).

Being unfamiliar with the system, coupled with computer literacy challenges were acknowledged to be contributors to the noted accessibility difficulties.

“… I think it is also a system so as he said, most of us we were born before computer so we were used to writing from school, exam, everything under pressure you write faster and with the computer, it’s a system so when you log in, you have to give time, the page will open, you do the next thing, you have to so it takes much time.” (GAR-NM 45).

#### Confidentiality

While others were concerned about difficulties in access, there were also contrary views that information in the EHR system was readily available for hospital personnel not directly involved in the patients' care hence, breaching confidentiality boundaries. The narratives below lay more emphasis.

“If I sit in surgical 4, I can check whatever is going on with the patients in another ward. So the issue of confidentiality is breached” (KBT-NM27); “ … So privacy is a big issue”. (GAR-NM54).

### EHR system training

From the theme “training provided on the EHR system”, four sub-themes emerged: (1) adequate and inadequate training, (2) the specific focus of training, (3) Transitioning challenge, and (4) involvement of health professionals in the development of the program was discussed.

#### Adequacy of training

Some health participants expressed feeling adequately trained on the EHR while others felt the training was inadequate.

“… we have had enough training but the whole thing boils down to individual attitude; do you have enough time to sit down to document? … because of the medico-legal issues, you have to think about writing everything that you do even whatever the patient says find time to write it, you can even quote the patient’s words because it’s necessary …” (GAR-AD8).

Inadequate training was indicated by the inability to apply knowledge from training and the constant need to request information technologist (IT) help, on issues that were covered in training.

“I think with the training we don’t have enough training because even during work, some people keep calling on the IT guys for petty issues they could have solved if they had adequate training.” (PH-NM23).

#### Specific or general training

Participants believed that there were variations in training programs that facilities were offered, others had training more specific to their departmental needs, while others received general training, which contributed to difficulties in their application to their units. Most participants from the middle and northern zones said they had received specific training as it was done according to the various departments.

“in the emergency unit, because we engage in health consultations of patients, we were additionally trained to use the consultation applications in the system…” ( BN-NM15).

Others stated that they were just given a general overview of the system not specific to their units.

“…It’s not customized…we were all lumped up for the training so maybe if it is done per unit then they will train us department by department.” (KBT-NM26).

#### Transitioning challenge

Participants in this study also complained about transitioning challenges. They were used to the manual system and are finding it difficult to adapt to the new system. The major cause of this challenge was the older age of some of the leaders especially, in the southern zone. For participants in the northern zone, this challenge was usually reported by participants who were not conversant with the computer system and were reluctant to learn.

“… transitioning from the paper to the paperless was a bit difficult for some of my colleagues, why? Because you need to almost type everything and some of us, don’t even remember the last time we did that. … (ASA-PA).

“ …it will be difficult for some of us to now learn these computer things (giggles). Let the younger ones who are interested learn we will work and let them d the documentation (laughing).” (Bawku -NM54).

#### Lack of end-user involvement in the development of EHR system

Participants verbalised that they are in separate positions with the team that works on the EHR system. For example, they lamented being excluded in discussions regarding the implementation of the system, including the lack of consideration of their inputs by the IT team, during the pilot phase of implementing the EHR, system in their hospitals. Additionally, in the pilot phase when suggestions were being given in some facilities, the IT team did not accept their input.

“And they don’t take suggestions…” (KBT-AC13).

“…with the current system I mean there are certain portions that are not supposed to be there, there are certain things that they are supposed to add too which are not there so I think if they are to consult us before even inputting those into it will be better.” (Sampa-NM42).

### EHR and workflow/work process

Some participants were in support of the EHR as it had drastically reduced waiting time, especially in the Records unit within the Out-Patient Department (OPD). Those (physician Assistants, medical assistants, and medical doctors) in the consulting rooms at the OPD, however, expressed displeasure with electronic entry because it slows them down. From this theme five (5) sub-themes emerged: (1) adequacy of computers, (2) standardisation of EHR across units, (3) network challenges, (4) Support from the IT team and (5) overall satisfaction with the EHR system.

#### Adequacy of computers

Not all institutions had the computer hardware for installing the EHR in place. This negatively affected the workflow. For example, most participants from the three (3) regions echoed the inadequacy in the number of computers available to work with. Participants noted that the consequences of a lack of computers were a lack of efficiency as everyone waited for one computer.

“Currently…, we have about 6 computers. 5 in the consulting rooms, and 1 in the vital sign's area but if we get an extra machine, we could separate the children and the aged so that they will go separately, yes but in the current situation, even if you separate them from the queue and you check them, we all come back to one machine before it goes to the prescriber’s end so if they add an extra one to it we will be very grateful”(GAR-NM22).

In an attempt to cope with the lack of computers, some had to bring personal laptops while other staff resorted to writing on paper while they wait in a queue to make entries on the computer. Additionally, some facilities installed the system on the phones of nurses to minimize pressure on available laptops.

“… being the head nurse, I must get a computer for myself and then read through what has been done. So that as I read through, I correct the errors, sometimes I don’t get any computer because the nurses are waiting for the doctors to finish their reviews and then they will do their changes. So which computer am I going to use to also go through the notes? Unless I bring a personal one” (Bono-NM35).

“…then it has been put on our phones as well so now when you enter the ward you see every nurse with a phone is not as though we are browsing… the doctors and the nursing staff we do” (KBT-NM12).

#### Lack of standardisation of EHR across units

Most participants complained that the EHR system in the facility is not standardized. In some instances, they suggested that some health facilities used much friendlier and simpler brands of the EHR system comparatively.

“…there is a health facility not far from us their system is better than ours. It is easier to navigate and they have standardized items for all units. I don’t know if it is the cost involved that is why our facility is not changing to that one…” (GAR-NM30).

#### Network challenges/unreliable

Participants also expressed challenges contributed by the instability of network and electricity fluctuations, especially during data entry and searches.

“… internet connection here is not one of the strongest and this is a huge challenge… you complete documentation and waiting to save the information. This takes ages or tells you system error then you have to start the process all over again” (KBT-NM44).

… whiles working on a client the light goes off you lose everything, or the system jams you find it very difficult, it takes a while before you can reconnect so it also slows down work” (GAR-NM13).

#### Support of IT team

In most facilities, participants expressed satisfaction with the IT team because they were swift and always available when needed, especially during the daytime.

“… yes they are available 24/7 we don’t have any problems with them. They respond quickly” (GAR-QA5).

However, the major problem was with issues encountered during the night shift, as there were no IT team assigned for the night.

“Sometimes the system can even go off for hours, we must wait for the IT personnel… and it affects client care … we’ve had issues with the system freezing in the middle of the night and we have to wait till the next day and clients will not go home, they are waiting to be taken care of”. (PH-NM3).

#### Overall satisfaction with the EHR system

Generally, the participants had differing expressions of satisfaction with the EHR. Most participants expressed dissatisfaction with the EHR.

“… I’m sure about 80% of the staff are all tired of the current system we are using because we’ve had challenges over and over again and it looks like when they try to rectify one challenge, another one pops up, so we are all tired…I will say poor” (GAR-NM24); “Very, very dissatisfied” (KB- NM4); “… yes it is making work difficult because doctors make inputs and sometimes it does not reflect at our end” (KB- PM7).

However, some participants were satisfied with the system.

“… satisfied, because it has reduced working time, referral information, in fact excellent” (HF, LH3); “… we are satisfied, yes we are satisfied. We pray that everybody will learn it and those who have refused to ‘click will click’, amen (general laughter)” (GAR-AD2).

Yet there were a few indifferent participants.

“neither here nor there, just as my colleague, because when the system is on it's very effective. We can work fast, but when the system is down too you can’t just enter anything and then you have to wait for it to come back before you can enter what you have done so yeah (BRH, NM4); “Though it is better compared to the manual system, there's more room for improvement”. (HI).

However, most participants across the country commended the introduction of the EHR system.

“…I want to add that apart from reducing the wait time, it has also reduced misfiling, so patient data is not lost though the new one is not detailed.” (BK-QA1).

## Discussion

EHR has now assumed an important role in healthcare delivery globally. It has therefore become imperative to assess the quality of care of EHR to ensure effective patient care and health service delivery. Health facilities in Ghana are using the EHR at various stages: pilot(one unit), a few units and all departments. Of the selected health facilities, three (3) were in the pilot phase where only the Outpatient Department (OPD) used it. However, the other six (6) facilities had fully implemented the EHR in all other units. The main aim of rolling out the EHR in the country was to improve the workflow process and quality of care in all healthcare settings.

In the current study, the researchers looked at the quality and satisfaction of health leaders with the EHR system from the end-user point of view. The findings on incomplete and inaccurate records are worrying as they indicate the incompetency of the users or lack of attention to detail during data capturing. Human resource and attitudinal barriers [2] such as laziness in the entry of client data, inaccurate entries and not being conversant with the electronic system, were some reasons for the poor quality of the EHR system.

The noticeable finding from this study was the fact that issues of confidentiality were raised in some facilities as policies on the protection of personal information are non-existent or not known. This confirms the findings of Gyamfi et al. [13] on barriers and facilitators to electronic medical records use in the emergency centre. Nonetheless, participants from some hospitals in the current study opined that they had resolved confidentiality issues with a stricter system and hence people who were outside a particular unit could not easily access the unit's EHR system. This could bring about ethico-legal issues in patient care so all health facilities should adopt stringent measures to maintain the privacy and confidentiality of patients’ data.

Interactions with the health leaders revealed that the training received was not adequate and also not tailored to their units in most cases. This agrees with the findings of Gui et al. [12],Parks et al. [21] and Pirtle et al. [22]. They advocated for regular in-house refresher training specific to units as this will make end users better equipped to adopt the EHR system.

With the influence of the EHR on workflow, participants in the outpatient department were grateful for the fast flow of patients from the records units to the nurses’ station where vital signs are checked. The challenge was mainly when patients move to the consulting rooms. The physician assistant in this study, confirmed studies by Stehman et al. [25] and Barrett [3] where physicians complained that the EHR slowed them down and sometimes caused depression. Additionally, the inadequacy of computers was reported as a challenge in all facilities and contributed to the delay in workflow/process. This confirms, a review by Katurura and Cilliers [16] and other empirical studies [19, 27] in developing countries. The financial constraints in some facilities and the bureaucratic processes in others could be the reason for this challenge. Thirdly, as Ghana is a low-middle income country, issues of network challenges related to the unreliability of electricity and internet were seen to hinder workflow when using the EHR. This confirms results from Zayyad and Toycan [27], who conducted an exploratory survey on the adoption of e-health technology in Nigerian hospitals from the perspective of healthcare professionals. To effectively and efficiently use the EHR system, measures need to be put in place for back-ups with both electric power and internet. This requires substantial and sustainable investments in information and communications technology (ICT) and electricity backup by hospital management.

With regards to satisfaction with the EHR system, youthful health leaders (below 45 years) in one of the health facilities were very satisfied with the EHR system and had also come up with innovations to make the system better (Nursemid). However, most participants across the three regions especially, the tertiary hospital, were not satisfied with the EHR system and were reluctant to commit to its use. This could likely be attributed to the generational differences among most participants as postulated by McCrorie et al. [17] which cause unacceptance of technology [27]. Considering most health leaders in this study were middle adults (45–55 years) this finding is not surprising. Older health leaders are used to the manual way of entering patient data and a little hitch in the electronic system deters their acceptance of the EHR system. Additionally, as confirmed by Adedeji et al. [2] and McCrorie et al. [17], the health leaders in this study, lamented that they were not involved in the development of the EHR. This also contributes to the non-acceptance stance of the health professionals in most facilities. Therefore, a higher number of younger health professionals who are willing to use the EHR need to be involved in software development and implementation to improve the quality of care for patients [18, 26].

## Limitations and strengths

Some limitations observed in this study include the fact that some of the initially selected facilities in some regions had to be excluded because they had not implemented the EHR system, and this affected the representation of private and quasi-health facilities in two regions. Additionally, medical doctors were hardly available during the focus group discussion so only one physician assistant represented the doctors. Since data werecollected from focus group discussions, participants' views may have been influenced by those of other group members. However, this study has several strengths, including the fact that it is one of the first studies to explore the views of the health leaders regarding the implemented EHR system, in the selected hospitals of Ghana. A major strength of this study is that participants were from various parts of the country and worked in facilities (public, private, quasi) which are representative of all the types of healthcare available nationwide. A review of the policy to involve the end users in the development and implementation process of the EHR system is expected to improve acceptability and healthcare provision among health professionals. Secondly, this when done will improve the quality of care provided to patients as well as the EHR system satisfaction of health professionals. It is hoped that further evaluation research can be conducted after the recommendations of the health leaders as suggested above have been implemented to identify differences in EHR satisfaction.

## Conclusion

We discovered that the health facility core management was involved in the setting up or choice of the EHR system however, the unit heads or frontline leaders especially nurses were the users or implementors of the system. Hence they must work hand-in-hand for maximum benefits. Hospital management in Ghana and most developing countries should be ready to invest in good EHR systems as well as regular power supply to successfully implement the EHR system. Secondly, they should liaise with the users of the EHR for the choice and installation of the system to improve satisfaction and get the best out of the EHR system. The study has also contributed to allowing advocacy for resources such as computers and generator plants as the researchers went back to present the findings to the hospitals. The findings of this study provide useful information that the implementation of an EHR system requires the involvement of frontline implementers in planning, their support with training is crucial, moreover, competency needs to be prioritized during training, to eliminate challenges associated with poor quality of records.

## Data Availability

The datasets used and/or analysed during the current study are available from the corresponding author on reasonable request.

## Abbreviations

EHR: Electronic health records
EHRs: Electronic health records system
FGD: Focus group discussion
IT: Information technology
OPD: Outpatient Department
PI: Principal Investigator
NM: Nurse manager
PM: Pharmacy Manager
AC: Accountants
MA: Medical Assistant
QA: Quality assurance
LH: Laboratory heads
AD: Administrator
HI: Health information officer
PA: Physician Assistant
GAR: Greater Accra Region
KBT: KorleBu Teaching Hospital
ASA: Amasaman Hospital
PH: Pentecost Hospital
BN: Bono
BK: Bawku
